# Digital pathology and artificial intelligence will be key to supporting clinical and academic cellular pathology through COVID-19 and future crises: the PathLAKE consortium perspective

**DOI:** 10.1136/jclinpath-2020-206854

**Published:** 2020-07-03

**Authors:** Lisa Browning, Richard Colling, Emad Rakha, Nasir Rajpoot, Jens Rittscher, Jacqueline A James, Manuel Salto-Tellez, David R J Snead, Clare Verrill

**Affiliations:** 1 Cellular Pathology, Oxford University Hospitals NHS Foundation Trust, Oxford, UK; 2 NIHR Oxford Biomedical Research Centre, Oxford, Oxfordshire, UK; 3 Nuffield Department of Surgical Sciences, Oxford University, Oxford, Oxfordshire, UK; 4 School of Medicine, University of Nottingham, Nottingham, Nottinghamshire, UK; 5 Tissue Image Analytics Laboratory, Department of Computer Science, University of Warwick, Coventry, West Midlands, UK; 6 Department of Pathology, University Hospitals Coventry and Warwickshire NHS Trust, Coventry, West Midlands, UK; 7 Department of Engineering Science and Big Data Institute, Oxford University, Oxford, Oxfordshire, UK; 8 Precision Medicine Centre of Excellence, Patrick G Johnston Centre for Cancer Research, Queen’s University Belfast, Belfast, Belfast, UK; 9 Cellular Pathology, Belfast Health and Social Care Trust, Belfast, Belfast, UK

**Keywords:** pathology, surgical, computer systems, image processing, computer-assisted

## Abstract

The measures to control the COVID-19 outbreak will likely remain a feature of our working lives until a suitable vaccine or treatment is found. The pandemic has had a substantial impact on clinical services, including cancer pathways. Pathologists are working remotely in many circumstances to protect themselves, colleagues, family members and the delivery of clinical services. The effects of COVID-19 on research and clinical trials have also been significant with changes to protocols, suspensions of studies and redeployment of resources to COVID-19. In this article, we explore the specific impact of COVID-19 on clinical and academic pathology and explore how digital pathology and artificial intelligence can play a key role to safeguarding clinical services and pathology-based research in the current climate and in the future.

## Cellular pathology services in the clinical and academic setting before the COVID-19 pandemic

Pathology is a vital part of cancer and other diagnostic pathways, being a core component in 70% of clinical interventions.^1^ Prior to the COVID-19 pandemic, cellular pathology (histopathology) capacity was not keeping pace with year-on-year increases in demand. The 2016 Cancer Research UK (CRUK) report ‘Testing Times to Come’ highlights increasing demand due to increasing cancer incidence, an ageing population and efforts to improve outcomes. Capacity has been further compounded by factors such as a preponderance of near retirement age pathologists.^2^ The CRUK report also describes the impact on pathology research with pathologists deprioritising this to focus on clinical work. The deprioritisation of research may become more acute as we see a resumption of clinical diagnostic and therapeutic services as the number of COVID-19 cases declines, with the subsequent increase in demand within our diagnostic laboratories. Digital pathology (DP) is seen as a key tool to enable more efficient use of the current workforce and digital enabled care is a core component of the National Health Service (NHS) long-term plan.^3^ In addition, the UK Government’s Industrial Life Sciences Strategy highlighted pathology as being ripe for innovation by the use of DP and artificial intelligence (AI).^4^

The PathLAKE consortium is one of the UK government’s 5 AI centres of excellence established in 2018 and funded by UK Research and Innovation (UKRI). PathLAKE features university and NHS partners from Coventry, Warwick, Belfast, Nottingham and Oxford. The aims include the creation of fully digital cellular pathology laboratories, the creation of an ethically approved datalake of anonymous scanned slide images and development of AI algorithms. The NHS laboratories have all now implemented DP technology as part of the programme, for example, with Oxford now scanning all slides generated in the diagnostic lab. The platforms have provided real time solutions to issues faced over the COVID-19 pandemic^5^ and will continue to do so with social distancing and other measures to contain the virus likely to be with us for some time.

## COVID-19 and clinical and academic cellular pathology

In March 2020, WHO characterised COVID-19 as a pandemic. Clinical services have seen significant alterations during this outbreak due to the novel severe acute respiratory syndrome-coronavirus (SARS-CoV-2) virus, and there have been logistical issues to consider in order to maintain cellular pathology services. Cancer pathways have been partly suspended or altered to reflect new risk–benefit profiles when determining how to treat and who to treat (and when). Medical and surgical societies, national and international authorities have issued revised guidelines on cancer care and the UK was one of several countries to suspend elective surgery during the worst parts of the crisis.^6^ This, together with the temporary cessation of national screening programmes has affected the volume and types of samples entering cellular pathology laboratories with direct consequences for research and training. Research programmes and clinical trials were paused or limited to only the most essential—in many institutions either those that are COVID-19 related or that would directly affect clinical care. As many institutions seek to reinstate some activity and on-site working, the challenges around maintaining social distancing in laboratories are significant. Levels of future funding for research due to reduced charitable income are also now in doubt.

Alongside the logistical issues with maintaining cellular pathology services during the pandemic, when a ‘new normal’ service begins, patterns of patient presentation, clinical prioritisation and volumes of work are likely to have changed. Modelling for cancer surgery delays and disruption for up to 2 years due to COVID-19 have, for example, predicted that a 6-month delay to cancer surgery across the population of the UK would result in 10 760 attributable deaths.^7^ As cancer pathways start to reopen, it is anticipated that those who did not seek help due to fears of burdening health services or catching this novel coronavirus in hospitals will present at a more advanced stage than they may have otherwise.^8^ A strategic push for early diagnosis in cancer will almost inevitably have been hampered by these factors.

In this article, we present the argument for DP and AI playing a key role in resilience for both academic pathology and diagnostic pathology services and that these should form an essential component of future planning. The article does not seek to cover cytology or postmortem practice, which have their own separate considerations. The issues are discussed in the context of the UK, but many will be applicable worldwide.

## Digital pathology and routine pathology services

For diagnostic purposes, the term ‘DP’ relates to the viewing on a computer workstation of digital whole slide images taken from high resolution scans of glass slides.^9^ Referred to as ‘the third pending revolution in pathology’,^10^ the benefits of DP as a potential solution to long-term workforce issues and the quality benefits are well described.^9 11^ DP represents an attractive option to increase resilience within often small and highly specialised teams, as well as departments, pathology networks and across the NHS. It can enable pathologists who are shielding or in self-isolation to continue to participate remotely in diagnostic services. In our practice, we have seen real-time solutions to workforce issues due to COVID-19.^5^ For example, digital double reporting and reducing transmission risk to remaining pathologists when several team members are in isolation. Pathologists who were previously reluctant to transition to DP are now supportive of fast adoption of the technology and in many cases, are the ones championing their way through the process. Within a wider context, several External Quality Assurance schemes are moving to full digitisation and removing glass slide circulations.

DP with entirely digital workflows including scanned request forms enables secure efficient remote working solutions, avoiding the need to transport glass slides and the inherent logistical problems and data governance risk this poses. Digital has health and safety benefits, with no physical movement of slides, avoiding potential for damage or (within this current crisis) infection transmission.

Reported time efficiency savings with DP^12 13^ will be important if labs are to cope with an anticipated backlog of cases due to deferment of surgery during this crisis period. Although the potential efficiencies are only likely to be realised with full digital deployments, smaller deployments of technology can provide solutions to specific problems to enable service continuity or may work for particular teams within a department without digitisation of the entire department. DP opens the door to other ways of reorganising work such as outsourcing cases to well-staffed departments and facilitating access to expert opinion, with some of these expert pathologists reporting from home. Frozen section diagnosis is another specific solution that can be provided by DP remotely. Seemingly overnight, healthcare institutions have rapidly adapted to this crisis, bringing in technologies such as video conferencing to allow team meetings and multidisciplinary team (MDT) or tumour board meetings. And thus, while the advice remains to work at home if at all possible, it would seem that there is little that the surgical pathologist would need to attend the hospital for, beyond cut up—and this can in many circumstances be undertaken by biomedical scientists.

With digitisation, the ability to run AI tools that can support pathologists by pre-screening, expediting workflow or providing a double check of the case is starting to become a reality.^14^ There are several AI tools on the ‘roadmap’ to full diagnostic use and some have regulatory clearance for such use, for example, in prostate cancer detection.^15^ With further evidence, AI could provide double reporting such as has been outlined as a possibility in mammography screening,^16^ providing resilience to services when pathologists are not available, and creating further efficiency gains. Collation of evidence to support and validate such tools does however require a digital workflow, and pathologists with time and facility to support such validation processes and integration of such tools into the routine pathology workflow.

Professional bodies play a key role in revisiting guidance and issuing urgent situation specific guidance. The Royal College of Pathologists issued ‘Guidance for remote reporting of DP slides during periods of exceptional service pressure’ in March 2020.^17^ This has described a risk mitigation approach to support temporary remote reporting with DP in this time of clinical and service necessity. The risk mitigation approach describes that primary diagnosis, double reporting, MDT review or quick review to determine if additional tests are needed each entail a different level of risk. And this needs to be considered alongside pathologist confidence and whether they been through formal DP validation. The College of American Pathologists have issued remote sign out guidance, including the use of digital platforms and concluded that remote working would ensure pathologist’s professional interpretations and diagnoses would continue during the crisis. Denying remote access to expert pathologists to provide the most accurate diagnoses would have potentially serious repercussions long after the current crisis.^18^

## Digital pathology and research

Many of the described advantages around flexibility and resilience in DP also apply to research and clinical trials.^19^ Restrictions to the research environment have been experienced during the COVID-19 pandemic. Standard of care guidelines have changed, limiting availability of samples and sample types for research. This affects not just cancer research, but any type of research where patients or samples are needed. Transplantation research, for example, may be disrupted by travel restrictions.^6^ The previous push for formalin free theatres to increase the availability of fresh samples for DNA/RNA/whole genome sequencing is now extremely challenging in view of the potential infection risk associated with the collection and processing of fresh tissue. While to date there are no reports of laboratory-acquired infection with SARS-CoV-2, this has been previously reported with SARS-CoV, although only in those labs in which the virus is propagated.^20^ Several guidance documents are now available addressing the handling of fresh or unfixed histopathological specimens using a risk-based approach to samples, including that which been published by Public Health England.^20^

Researchers who would normally use fresh or fresh frozen material may therefore have to defer to using formalin-fixed paraffin-embedded material where virus is thought to be inactivated and fixation may need to be prolonged, further damaging DNA. This may be less optimal, for example, in whole genome sequencing where high quality nuclear acids are required. Biobanking has looked quite different during the COVID-19 pandemic in some instances,^21^ for example face-to-face consenting, the previous norm, often replaced with remote consent procedures such as over the telephone with virtual signature.

Several research articles covering the interplay between cancer and COVID-19 have been published, many from China and Italy, and they focus mainly on four areas: the effect of cancer therapy in COVID-19, epidemiological/clinical/radiological/pathological features, outcomes of patients with cancer with COVID-19 and strategies for risk reduction and management.^22^ According to an analysis of 355 Italian patients, 20% of those who died from COVID-19 had active cancer.^23^ However overall, further evidence for the effect of COVID-19 on patients with cancer are needed.^24^ Pathologists are key here in helping to describe the changes in this essentially new disease and DP enables cases to be shared with the leading experts in the field.^25–27^ The Worldwide DP Alliance has established a global resource of postmortem whole slide images aimed at supporting understanding of the pathology of this new disease and accelerating access to samples for research.^28^ There are also new online image repositories being established by the National Institutes of Health and RCPath.^29 30^

As cohorts build, existing expertise in AI can be repurposed to search for novel features. As these resources grow to the cohort sizes usually associated with AI, they will become extremely valuable. Setting these resources up and ensuring the appropriate governance and data sharing arrangements are in place may pave the way for future joined up working and the potential reduction in ‘wastage’ of research resources. Given the impact that post-mortem research had in the previous SARS Co-V pandemic^31^ this concept of DP/AI facilitating the use of material for research purposes is important and may go some way to addressing the current concerns of autopsies not playing a vital role in COVID-19 research.^32^

AI lends itself well to interpretation of immuno-oncology sections stained for multiple immune markers either on serial sections or by multiplex technology, establishing novel insights into cell subpopulation densities and topographical relationships between cells and other structures. This kind of expertise can be quickly repurposed to support similar studies of immune populations in COVID-19.^33 34^ Another example of the redeployment of AI expertise is the use of machine learning to improve the efficiency of COVID-19 testing, ensuring optimal use of resources.^35^

We have seen the disruptive effect of the COVID-19 pandemic on clinical trials, with immediate and delayed consequences. Clinical trial staff have in many cases been redeployed to other clinical areas and many clinical trials have been suspended to recruitment with trials into COVID-19 being prioritised. There has been disruption to almost every aspect of trial process, which may require protocol deviations. Examples include, the inability to conduct participant protocol-mandated visits, staff training, site monitoring visits and interruptions to supply chains of drugs. These interruptions may result in overall delays to trial delivery and drug development and COVID-19 deaths may potentially disrupt the endpoints of trials. Urgent guidance has been issued by the US Food and Drug Administration and the European Medicines Agency on clinical trial conduct during the pandemic.^36 37^

Clinical trials may be more heavily affected by COVID-19 in particular settings, for example in some areas of oncology practice. In haematology oncology, patients are often immunosuppressed due to illness or treatment or both and are highly susceptible to severe complications if infected with COVID-19^38^ leading to a serious and disruptive effect on haematology oncology trials.^24^ Patients with lung cancer represent another highly vulnerable group as other pre-existing pulmonary diseases such as chronic obstructive pulmonary disease, cardiovascular disease and old age contribute to the effects of the virus.^38^ The effects on immunotherapy trials and the complex potential interplay between such agents and COVID-19 is currently the subject of debate.^39^

As pathologists, we deliver and support clinical trials, and quality assurance of the pathology input is key to assessing baseline tests and endpoints. Quality is determined by setting up study specific pathology working groups, training and standardisation events and double/central reporting of parameters.^40^ All of these are now logistically challenging, but this is where DP provides a timely opportunity to enable these to continue remotely and may accelerate the move towards this model of working in the future.^41^ Other vital clinical trial activities such as block selection can be easily done remotely by DP. Trial monitoring by regulators could also still be enabled although data sharing restrictions may apply. We should expedite opportunities to use AI to assess trial endpoints. AI makes better use of pathologists’ time and standardises assessments, such as scoring or grading that might traditionally have been undertaken by pathologists with inherent problems of human observer subjectivity.

The availability of tissue samples on which to assess exploratory trial endpoints may be reduced by optional study-specific biopsies not being undertaken. Logistical challenges around different geographic locations, for example, the use of COVID-19 free cancer hubs or clinical trial sites being unavailable may mean that alternative laboratories may be analysing samples. If this occurs, DP presents an opportunity to move images around to the relevant trial pathologists with the necessary expertise or to enable AI interpretation. This ensures standardisation of baseline tests such as biomarker test for entry, or interpretation of primary or secondary endpoints.

## Digital pathology and education

Another important application of DP that can be exploited at this time is in the maintenance of education and training. With consultant histopathologists encouraged to work remotely, it is easy to imagine that those in training may consequently find it more challenging to review clinical cases with a consultant, potentially impacting on training opportunities. The importance of this has been noted by other recent authors^42^ who identify DP as a means of access to clinical cases for continued training of pathologists during this period. Indeed, this is the case within our own practice whereby face-to-face contact with our trainees has significantly reduced outside of the cut-up room. We have found that DP is of benefit in this setting, with trainees able to access digitally and discuss cases remotely with supervisors, either live through screen sharing on a secure videoconferencing facility, on the telephone, or through email exchange, with trainees and consultants annotating or manipulating digital slides live. One suspects that contrary to expectations of this having a negative impact on learning, that being able to focus very specifically on certain annotated details may actually improve the educational value of reviewing cases. Dedicated teaching sessions have been undertaken by our teams through the use of secure videoconferencing facilities, opening the opportunity to teach across departments, and to reach out to those unable to attend the workplace. While these measures show great potential, we must be mindful of the potential impact on training that working remotely may have and as trainers we need to consider ways to fully include trainees in our daily working patterns.

## Challenges

While the benefits of DP are apparent, the challenges are not insignificant. In addition to logistical challenges of setting up digital pathology services within the diagnostic laboratory, there are challenges further down the line with the development and exploitation of ancillary tools such as AI. These include the regulatory path to use of AI, public perception of data sharing, and the many technological challenges, including lack of a standard image format. We must learn lessons from other healthcare settings, build trust in these new technologies and encourage routine adoption. For example, video consultations have been adopted by hospital doctors and general practitioners to reduce face-to-face interactions,^43^ but several factors have had to be considered in order to scale this technology such as improved dependability and reasonable cost.^43^ Reported issues which delay uptake in this setting include inappropriate platforms with potential for data breaches and lack of bandwidth. It must be clear that change cannot be achieved by simply deploying technology. We are introducing and sustaining major changes to a complex system, which we need to acknowledge is often difficult, can be resource intensive and needs to be championed by respected opinion leaders.^43^

We should also share experience with our colleagues working with other applications of AI in medical sciences, for example, the imaging community who have been working with AI longer than those in the pathology community. AI has, for example, been used in radiology in order to diagnose chest X-ray images as COVID-19 with an overall accuracy of 89.6% when compared with normal or pneumonia (bacterial or viral)^44^ and quantify opacification on lung CT in order to determine clinical severity of COVID-19.^45^

## Conclusion

We have described the great potential of digital technologies in pathology, and how DP can provide immediate solutions to service delivery, research, clinical trial and training issues in a pandemic crisis. With no instant exit from the current measures that are in place to reduce transmission of COVID-19, DP is likely to continue to prove its worth in providing the necessary flexibility and resilience for cellular pathology. Pathology networks and academic departments should be encouraged to evaluate where the technology fits into their contingency plans. Although adoption of such technology was already being encouraged, COVID-19 has fortuitously pushed DP into the spotlight. Moreover, we can learn from the implementation of DP in these settings during this acute crisis, to allow us to identify and champion its value, and to recognise any potential pitfalls, and with the necessary ‘hoops’ to implementation in these settings already resolved. The COVID-19 crisis has actually given us an opportunity to take advantage of providing, as with other aspects of healthcare, a catalyst to reorganise dated working practices within pathology, embracing the advantages technological advancements provide to deliver safer and more efficient services. However, let us not forget that the vast majority of cellular departments in the UK have limited or no access to this technology for diagnostic purposes.^46^ Now is the time to realise the potential of DP and for us to call for more investment in this technology. This should not only be investment in intradepartmental infrastructure but technological and infrastructural support for pathologists at home.

**Take home messages**

- The measures to control the COVID-19 outbreak will likely remain a feature of our working lives until a suitable vaccine or treatment is found.
- Digital pathology (DP) is seen as a key tool to enable more efficient use of the current workforce and digital enabled care is a core component of the National Health Service (NHS) long term plan.
- Digital pathology and artificial intelligence can play a key role to safeguarding clinical services, training and pathology-based research in the current climate and in the future.

## References

[1] Report of the review of NHS pathology services in England, a report for the Department of health, Chaired by Lord Carter of Coles 2006.

[2] CRUK . Testing times to come? an evaluation of pathology capacity across the UK, 2016. Available: https://www.cancerresearchuk.org/sites/default/files/testing_times_to_come_nov_16_cruk.pdf [Accessed 01 May 2020].

[3] NHS long term plan, 2019. Available: https://www.longtermplan.nhs.uk/online-version/overview-and-summary/ [Accessed 01 May 2020].

[4] Office for Life Sciences . Life sciences: industrial strategy. A report to government from the life sciences sector, 2017. Available: https://www.gov.uk/government/publications/life-sciences-industrial-strategy [Accessed 19 May 2020].

[5] Browning L , Fryer E , Roskell D , et al . Role of digital pathology in diagnostic histopathology in the response to COVID-19: results from a survey of experience in a UK tertiary referral hospital. J Clin Pathol 2021;74:129–32. 10.1136/jclinpath-2020-206786 32616541 PMC7841475

[6] Burki TK . Cancer guidelines during the COVID-19 pandemic. Lancet Oncol 2020;21:629–30. 10.1016/S1470-2045(20)30217-5 32247319 PMC7270910

[7] Sud A , Jones M , Broggio J , et al . Collateral damage: the impact on outcomes from cancer surgery of the COVID-19 pandemic. Ann Oncol 2020. 10.1016/j.annonc.2020.05.009. [Epub ahead of print: 16 May 2020]. PMC7237184 32442581

[8] Bowman R , Crosby DL , Sharma A . Surge after the surge: Anticipating the increased volume and needs of patients with head and neck cancer after the peak in COVID-19. Head Neck 2020. 10.1002/hed.26260. [Epub ahead of print: 16 May 2020]. PMC7276844 32415869

[9] Williams BJ , Bottoms D , Treanor D . Future-proofing pathology: the case for clinical adoption of digital pathology. J Clin Pathol 2017;70:1010–8. 10.1136/jclinpath-2017-204644 28780514

[10] Salto-Tellez M , Maxwell P , Hamilton P . Ai – the third revolution in pathology. Histopathology 2019;74:372–6. 30270453 10.1111/his.13760

[11] Williams BJ , Bottoms D , Clark D , et al . Future-proofing pathology Part 2: building a business case for digital pathology. J Clin Pathol 2019;72:198–205. 10.1136/jclinpath-2017-204926 29549217

[12] Hanna MG , Reuter VE , Samboy J , et al . Implementation of digital pathology offers clinical and operational increase in efficiency and cost savings. Arch Pathol Lab Med 2019;143:1545–55. 10.5858/arpa.2018-0514-OA 31173528 PMC7448534

[13] Retamero JA , Aneiros-Fernandez J , Del Moral RG , et al . Complete digital pathology for routine histopathology diagnosis in a multicenter Hospital network. Arch Pathol Lab Med 2020;144:221–8. 10.5858/arpa.2018-0541-OA 31295015

[14] Niazi MKK , Parwani AV , Gurcan MN . Digital pathology and artificial intelligence. Lancet Oncol 2019;20:e253–61. 10.1016/S1470-2045(19)30154-8 31044723 PMC8711251

[15] Colling R , Pitman H , Oien K , et al . Artificial intelligence in digital pathology: a roadmap to routine use in clinical practice. J Pathol 2019;249:143–50. 10.1002/path.5310 31144302

[16] Schaffter T , Buist DSM , Lee CI , et al . Evaluation of combined artificial intelligence and radiologist assessment to interpret screening mammograms. JAMA Netw Open 2020;3:e200265. 10.1001/jamanetworkopen.2020.0265 32119094 PMC7052735

[17] Royal College of Pathologists . Guidance for remote reporting of digital pathology slides during periods of exceptional service pressure, 2020. Available: https://www.rcpath.org/uploads/assets/626ead77-d7dd-42e1-949988e43dc84c97/RCPath-guidance-for-remote-digital-pathology.pdf [Accessed 19 May 2020]. 10.4103/jpi.jpi_23_20 PMC7245343 32477618

[18] College of American Pathologists . COVID19 – remote sign-out guidance, 2020. Available: https://documents.cap.org/documents/COVID19-Remote-Sign-Out-Guidance-vFNL.pdf [Accessed 11 May 2020].

[19] Hamilton PW , Bankhead P , Wang Y , et al . Digital pathology and image analysis in tissue biomarker research. Methods 2014;70:59–73. 10.1016/j.ymeth.2014.06.015 25034370

[20] Public Health England . COVID-19: safe handling and processing for samples in laboratories, 2020. Available: https://www.gov.uk/government/publications/wuhan-novel-coronavirus-guidance-for-clinical-diagnostic-laboratories/wuhan-novel-coronavirus-handling-and-processing-of-laboratory-specimens [Accessed 19 May 2020].

[21] Vaught J . Biobanking during the COVID-19 pandemic. Biopreserv Biobank 2020;18:153–4. 10.1089/bio.2020.29069.jjv 32297797

[22] Moujaess E , Kourie HR , Ghosn M . Cancer patients and research during COVID-19 pandemic: a systematic review of current evidence. Crit Rev Oncol Hematol 2020;150:102972. 10.1016/j.critrevonc.2020.102972 32344317 PMC7174983

[23] Onder G , Rezza G , Brusaferro S . Case-Fatality rate and characteristics of patients dying in relation to COVID-19 in Italy. JAMA 2020. 10.1001/jama.2020.4683. [Epub ahead of print: 23 Mar 2020]. 32203977

[24] Saini KS , de Las Heras B , de Castro J , et al . Effect of the COVID-19 pandemic on cancer treatment and research. Lancet Haematol 2020;7:e432–5. 10.1016/S2352-3026(20)30123-X 32339482 PMC7195053

[25] Kolivras A , Dehavay F , Delplace D , et al . Coronavirus (COVID-19) infection–induced chilblains: a case report with histopathologic findings. JAAD Case Rep 2020;6:489–92. 10.1016/j.jdcr.2020.04.011 32363225 PMC7194989

[26] Peleg Y , Kudose S , D'Agati V , et al . Acute kidney injury due to collapsing glomerulopathy following COVID-19 infection. Kidney Int Rep 2020. [Epub ahead of print: 28 Apr 2020]. 10.1016/j.ekir.2020.04.017 PMC7186120 32346659

[27] Pernazza A , Mancini M , Rullo E , et al . Early histologic findings of pulmonary SARS-CoV-2 infection detected in a surgical specimen. Virchows Archiv 2020;395. 10.1007/s00428-020-02829-1 PMC7192563 32356025

[28] The Alliance for Digital Pathology . COVID-19 worldwide digital Repository. Available: https://digitalpathologyalliance.org/covid19 [Accessed 19 May 2020].

[29] NIH . COVID-19 scientific interest group. Available: https://oir.nih.gov/sigs/covid-19-scientific-interest-group [Accessed 19 May 2020].

[30] The Royal College of Pathologists . RCPath COVID-19 post mortem portal. Available: https://www.rcpath.org/profession/coronavirus-resource-hub/covid-19-post-mortem-portal.html [Accessed 19 May 2020].

[31] Kong SL , Chui P , Lim B , et al . Elucidating the molecular physiopathology of acute respiratory distress syndrome in severe acute respiratory syndrome patients. Virus Res 2009;145:260–9. 10.1016/j.virusres.2009.07.014 19635508 PMC7114434

[32] Ledford H . Autopsy slowdown hinders quest to determine how coronavirus kills. Nature 2020. 10.1038/d41586-020-01355-z. [Epub ahead of print: 07 May 2020]. 32382121

[33] Rao SR , Alham NK , Upton E , et al . Detailed molecular and immune marker profiling of archival prostate cancer samples reveals an inverse association between TMPRSS2:ERG fusion status and immune cell infiltration. J Mol Diagn 2020;22:652-669. 10.1016/j.jmoldx.2020.02.012 32229180

[34] Koelzer VH , Sirininukunwattana K , Rittscher J , et al . Precision immunoprofiling by image analysis and AI. Virchows Archiv 2018;474:511–22. 30470933 10.1007/s00428-018-2485-z PMC6447694

[35] Minhas F , Grammatopoulos D , Young L , et al . Improving COVID-19 testing efficiency using guided agglomerative sampling. bioRXiv 2020.

[36] FDA . FDA guidance on conduct of clinical trials of medical products during Covid-19 public health emergency, 2020. Available: https://www.fda.gov/media/136238/download [Accessed 19 May 2020].

[37] European Medicines Agency . Guidance on the management of clinical trials during the COVID-19 (coronavirus pandemic) V3. Available: https://ec.europa.eu/health/sites/health/files/files/eudralex/vol-10/guidanceclinicaltrials_covid19_en.pdf [Accessed 13 May 2020].

[38] Fuereder T , Gunsilius E , Bartsch R , et al . Circumnavigating the challenges of COVID-19 in oncology. Memo 2020:1–4. 10.1007/s12254-020-00611-2 32382356 PMC7203542

[39] Bersanelli M . Controversies about COVID-19 and anticancer treatment with immune checkpoint inhibitors. Immunotherapy 2020;12:269–73. 10.2217/imt-2020-0067 32212881 PMC7117596

[40] Robinson M , James J , Thomas G , et al . Quality assurance guidance for scoring and reporting for pathologists and laboratories undertaking clinical trial work. J Pathol Clin Res 2019;5:91–9. 10.1002/cjp2.121 30407751 PMC6463860

[41] Pell R , Oien K , Robinson M , et al . The use of digital pathology and image analysis in clinical trials. J Pathol Clin Res 2019;5:81–90. 10.1002/cjp2.127 30767396 PMC6463857

[42] Roy SF , Cecchini MJ . Implementing a structured digital-based online pathology curriculum for trainees at the time of COVID-19. J Clin Pathol 2020;73:444. 10.1136/jclinpath-2020-206682 32366598

[43] Greenhalgh T , Wherton J , Shaw S , et al . Video consultations for covid-19. BMJ 2020:m998. 10.1136/bmj.m998 32165352

[44] Khan AI , Shah JL , Bhat MM . CoroNet: a deep neural network for detection and diagnosis of COVID-19 from chest X-ray images. Comput Methods Programs Biomed 2020;196:105581. 10.1016/j.cmpb.2020.105581 32534344 PMC7274128

[45] Huang L , Han R , Ai T , et al . Serial quantitative chest CT assessment of COVID-19: Deep-Learning approach. Radiology 2020;2:e200075. 10.1148/ryct.2020200075 PMC7233442 33778562

[46] Williams BJ , Lee J , Oien KA , et al . Digital pathology access and usage in the UK: results from a national survey on behalf of the National cancer research Institute's CM-Path initiative. J Clin Pathol 2018;71:463–6. 10.1136/jclinpath-2017-204808 29317516 PMC5916098

